# Implementation of the ISO 4037:2019 standard using voltage dividers and X-ray spectrometry

**DOI:** 10.1093/rpd/ncag059

**Published:** 2026-06-17

**Authors:** Luigi Rinaldi, Alessia Ciccotelli, Claudia Silvestri, Andrea Petrucci, Gianluca Cappadozzi, Susy Toma, Serena Fattori, Parvin Mohammadyari, Francesca Curciarello, Joonas Tikkanen, Paula Toroi, Massimo Pinto

**Affiliations:** Istituto Nazionale di Metrologia delle Radiazioni Ionizzanti, ENEA-INMRI, Centro Ricerche ENEA Casaccia, Via Anguillarese 301, 00123 Santa Maria di Galeria, Roma, Italy; Istituto Nazionale di Metrologia delle Radiazioni Ionizzanti, ENEA-INMRI, Centro Ricerche ENEA Casaccia, Via Anguillarese 301, 00123 Santa Maria di Galeria, Roma, Italy; Istituto Nazionale di Metrologia delle Radiazioni Ionizzanti, ENEA-INMRI, Centro Ricerche ENEA Casaccia, Via Anguillarese 301, 00123 Santa Maria di Galeria, Roma, Italy; Istituto Nazionale di Metrologia delle Radiazioni Ionizzanti, ENEA-INMRI, Centro Ricerche ENEA Casaccia, Via Anguillarese 301, 00123 Santa Maria di Galeria, Roma, Italy; Istituto Nazionale di Metrologia delle Radiazioni Ionizzanti, ENEA-INMRI, Centro Ricerche ENEA Casaccia, Via Anguillarese 301, 00123 Santa Maria di Galeria, Roma, Italy; Istituto Nazionale di Metrologia delle Radiazioni Ionizzanti, ENEA-INMRI, Centro Ricerche ENEA Casaccia, Via Anguillarese 301, 00123 Santa Maria di Galeria, Roma, Italy; Dept. of Electrical and Information Engineering, University of Cassino and Southern Lazio, Cassino, Italy; Istituto Nazionale di Metrologia delle Radiazioni Ionizzanti, ENEA-INMRI, Centro Ricerche ENEA Casaccia, Via Anguillarese 301, 00123 Santa Maria di Galeria, Roma, Italy; Istituto Nazionale di Metrologia delle Radiazioni Ionizzanti, ENEA-INMRI, Centro Ricerche ENEA Casaccia, Via Anguillarese 301, 00123 Santa Maria di Galeria, Roma, Italy; Istituto Nazionale di Metrologia delle Radiazioni Ionizzanti, ENEA-INMRI, Centro Ricerche ENEA Casaccia, Via Anguillarese 301, 00123 Santa Maria di Galeria, Roma, Italy; Radiation and Nuclear Safety Authority, STUK, Jokiniemenkuja 1, Vantaa, Finland; Radiation and Nuclear Safety Authority, STUK, Jokiniemenkuja 1, Vantaa, Finland; Istituto Nazionale di Metrologia delle Radiazioni Ionizzanti, ENEA-INMRI, Centro Ricerche ENEA Casaccia, Via Anguillarese 301, 00123 Santa Maria di Galeria, Roma, Italy

## Abstract

The ISO 4037:2019 standard is the reference standard for dosimetry laboratories who wish to realize dosimetric operational quantities for radiation protection calibrations. In implementing the ISO 4037:2019 standard, the X-ray radiation qualities need to be defined according to strict requirements on the material and thickness of the additional filtration, and according to metrologically traceable high voltage bias applied to the X-ray tube. This enables usage of standardized conversion coefficient from air kerma to operational quantities. However, the tube potential may vary as a function of tube current if a protective resistor is built into the protective tube housing, which, if not corrected, alters the energy distributions of the reference field and thus the value of the appropriate conversion coefficients. Particularly at low energies, the energy dependence of the conversion coefficients can be sharp and, depending on the specific realization of a radiation quality, the conversion coefficient from air kerma to dose equivalent can vary substantially. We have investigated the correspondence between the tube voltage measurements of a X-ray system based on calibrated voltage dividers and that of an X-ray spectrometer system based on a Cadmium Telluride (CdTe) detector in order to obtain an estimation of the resistance of the protective resistor that is usually unknown. A method based on X-ray spectrometry for calibration of the tube potential even in the presence of tube with protective resistor is presented. Finally, conversion coefficients were calculated using simulated spectra to study the influence of the protective resistor on the determination of these coefficients. The simulated spectra obtained from X-ray tube with and without a protective resistor resulted in differences in conversion coefficients mostly ˂2% but 5.6% for the radiation quality considered at the lowest energy.

## Introduction

The realization of the current operational quantities in radiation protection dosimetry, e.g. the personal or ambient dose equivalent, currently relies on the realization of the air kerma and the use of calculated conversion coefficients to convert the physical quantity of air kerma to the dosimetric operational quantity of choice. The standard ISO 4037 [1–3] defines the criteria for the realization of radiation qualities in radiation protection dosimetry. These operational quantities are defined to provide a conservative estimate of the quantity effective dose, which cannot be determined experimentally. For most laboratories who may not have access to X-ray spectrometry techniques, the recommended implementation of the ISO 4037:2019 standard is that of ‘matched fields’ [1]. This requires tight control over both the filter material, purity and thickness, and the use of voltage dividers with a traceable calibration, the ISO recommended method to determine the voltage applied to the X-ray tube. The voltage dividers, however, must be used with caution as although they may, when calibrated, correctly indicate the voltage that is downstream to the high voltage supply, they may not indicate the voltage that is effectively available on the X-ray tube. In fact, the ISO 4037:2019 Part 1 in Section 4.2.2 states that the tube potential (potential difference between cathode and anode of the X-ray tube) is not always equal to the generating potential (the potential difference between the high voltage electrical connections of the protective housing of the X-ray tube). The former can be smaller, if a protective resistor is built into the protective tube housing, causing a voltage drop. In addition, the difference between these two voltages would then also depend on the tube current. It is important to know from the manufacturer of the protective tube housing whether such a protective resistor is installed. Unfortunately, information about the protective resistor is not always easy to obtain from the manufacturer and, if present, its exact value is unknown to the user. To overcome these difficulties, we propose to determine the potential difference between anode and cathode with a non–invasive method, by estimating the endpoint energy of an X-ray spectrum from the measured pulse-height spectrum. A measurement of the maximum photon energy (*E*_max_) with a spectrometer is performed, and the tube potential can be obtained with equation *V* = *E*_max_*/e*, where *e* is the elementary charge. Other authors proposed a spectrometric method for the determination of the spectrum endpoint energy based on the comparison of the shape of the end part of the measured detector spectra to those of the Monte Carlo simulated detector spectra [4]. This method requires that a Monte Carlo model of the detector must be available and validated, and charge carrier transport (CCT) plus Gaussian Energy Broadening (GEB) valid in the appropriate count-rate regime should be applied on the simulated detector spectrum. The ISO 4037:2019 standard mentions that the protective resistor is usually in the order of 100 kΩ. As an example, this means that, at a nominal tube current of 20 mA, the tube potential is reduced by 2 kV as compared to the generating potential. This reduction, if not corrected, alters the energy distribution of the reference field and may have an impact on the value of the appropriate conversion coefficients. Therefore, the tube voltage needs to be corrected to compensate for the voltage drop due to the protective resistor, when this is present. The voltage drop across this resistor shall then be determined in order to set the desired tube potential. After the publication of the ISO 4037:2019 standard, several laboratories faced the problems caused by the presence of the protective resistor on their tube which, if present in their installation, would make the acquisition of voltage dividers only half useful. Some laboratories just verify the presence of the protective resistor studying the linearity of the air kerma rate as a function of the current in the X-ray tube [5], and other authors use a high purity germanium (HPGe) spectrometer to acquire the pulse height spectrum and obtain the spectrum endpoint energy showing a variation in the voltage as a function of tube current [6]. In this work, we want to show the effects that the presence of the protective resistor produces and provide a method to estimate the value of the protective resistor using a CdTe spectrometer. The CdTe spectrometer is arguably more affordable than a HPGe spectrometer, as well as more practical because the small dimensions make transport and positioning in the beam relatively simple. Importantly, no spectrum unfolding procedure is needed for the endpoint energy estimation. The goal of unfolding is the reconstruction of the undisturbed fluence spectrum which would prevail at the location of the detector if the detector was taken away. To remove detector effects from a measured spectrum, the photon response of the detector has to be known as precisely as possible [7]. The shape of the undisturbed photon fluence spectrum is not important when the interest is in the endpoint energy of the radiation spectrum. This makes this procedure and the use of the spectrometer relatively easy, and a laboratory can apply this procedure without particular trouble in collecting and analyzing data. Furthermore, we want to propose a useful and accessible alternative solution for those laboratories that cannot afford the purchase costs of the voltage divider and its subsequent re-calibrations, which also can impact the running costs. Our study shows that the method developed in our institute, based on the measurement of highly filtered spectra using a CdTe spectrometer, allows the tube potential as a function of tube current to be calibrated on site even in the presence of a protective resistor in the X-ray tube. With the proposed method, knowing the shape of the fluence spectrum is not necessary, so there is no need to unfold the measured pulse-height spectra. However, knowledge of the spectrometer energy calibration, and the energy resolution, is necessary. To support our point, we have investigated the correspondence between the readings of a system based on calibration voltage dividers and that of an energy-calibrated X-ray spectrometer system based on a CdTe detector. In quantifying the differences, we estimate the uncertainties associated with the implementation of the ISO 4037:2019 standard when relying on the use of calibrated voltage dividers and when the effects of the protective resistor are not properly taken into account. Finally, an estimation of several conversion coefficients ${h}_K^{\ast}\left(10;N\right)$ is presented to quantify the implication of using a different voltage value due to the presence of the protective resistor in the X-ray system. The criticality of the issue is that if a laboratory is unable to purchase and maintain the voltage dividers, they do not comply with the current standard and the radiation protection calibration would not be recognized and accepted by regulatory bodies. The results presented here provide a basis for developing updated recommendations in this area.

## Materials and methods

### Irradiation facilities

Measurements were made in two metrology laboratories: the Italian National Institute of Ionizing Radiation Metrology (ENEA-INMRI), and the Secondary Standard Dosimetry Laboratory of the Finnish Radiation and Nuclear Safety Authority (STUK). ENEA-INMRI is the Italian primary standards dosimetry laboratory (PSDL) for ionizing radiation quantities. Concerning the kilovoltage X-ray energies, a fairly wide range of qualities are available for medical, industrial, and radiation protection purposes. In this study, measurements at ENEA-INMRI were carried out on a bipolar ISOVOLT (called ENEA-INMRI X-ray tube in this work) manufactured by GE and equipped with two GE 225 kV high voltage (HV) generators. Both high voltage generators are complemented by calibrated voltage dividers manufactured by WayGate Technologies. The X-ray tube has a built-in protective resistor. The divided voltage signal was read either by a high impedance calibrated electrometer or sent to an oscilloscope for analysis of the high voltage ripple. The calibration certificate of each divider was issued with Mutual Recognition Arrangement - International Laboratory Accreditation Cooperation (MRA-ILAC) compliance upon first purchase of the equipment. The secondary standards dosimetry laboratory of STUK is the Finnish designated metrology institute that maintains national measurement standards for ionizing radiation. The laboratory has two different industrial X-ray systems, a low energy tube GE, ISOVOLT TITAN 160 kV (called STUK low energy tube in this work) and a GE, ISOVOLT TITAN 320 kV (called STUK medium energy tube in this work). The STUK X-ray tubes do not have a protective resistor. There are no voltage dividers installed on the high voltage generators at STUK. However, a voltage divider was rented for a short period of time to get traceable voltage measurement for the STUK low energy tube. The constancy of the generator voltage output is checked regularly with HPGe spectrometry measurements, and during calibrations with a monitor chamber; deviations smaller than 0.1 kV in the tube voltage would be visible in the spectrum, and deviations in voltage higher than the limits given by ISO 4037 would cause a change in the monitor chamber reading higher than the tolerance set by the laboratory. The voltage determination for the STUK medium energy tube is based on spectrometry measurements only.

### X-ray spectrometer

The spectrometer used for this study is a commercial spectrometer X-123 CdTe (AmpTek/AMETEK). The electronics and cooling system together with the sensor are contained in a single small housing that fits in the palm of a hand. The spectrometer is equipped with a collimation system, including a hollow brass cylinder which can house several tungsten collimators inside. The aim of the collimation system is to reduce the fluence of incident X-rays to allow the electronics to process the spectrum by limiting the effects of pile-up of counts. The instrument was supplied with five tungsten collimators with nominal diameters ranging from 25 μm to 2000 μm. If a measurement of photon fluence was sought, a verification of the actual aperture diameters would be necessary, as in [5]. For our measurements, we used a stack made of three collimators with nominal diameters 2 mm, 1 mm, and 400 μm going from the X-ray tube to the detector entrance surface. The spectrometer is positioned in the beam and the collimator aperture is aligned along the direction parallel to the longitudinal beam axis with a laser pointer. For a measurement of endpoint energy, the correct alignment of the CdTe detector in the photon beam is not as crucial as it would be for the determination of the photon fluence spectrum. Any scatter from a misaligned collimator set would affect the shape of the fluence spectrum but not its endpoint energy.

### Energy calibration and detector energy resolution

**Figure 1:**
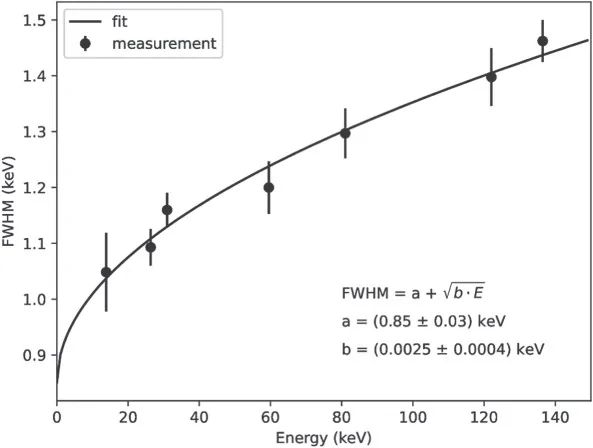
Fit of the measured energy resolution with CdTe detector.

The energy calibration of the CdTe spectrometer was achieved using low activity point sources from the radioactivity division of ENEA-INMRI. The sources used when calibrating the detector were ^241^Am, ^133^Ba, ^152^Eu and ^57^Co, and the calibration was checked at STUK with ^133^Ba and ^57^Co sources. The activities of these sources was chosen so as to keep the detector count rate below 10 000 *s*^−1^. For the energy calibration, the user is assisted by a purposely-developed Python script which offers an automated calibration procedure. Information on the energy ‘peaks’ to be sought using a CdTe type detector, for each nuclide, are stored in an ASCII file nuclide library which is consulted by the Python script to mark a correspondence between the centroid of an energy peak and its energy value. The user can apply such a procedure on the raw spectrum, i.e. with no need to apply any corrections toward the elimination of all distorting effects that are caused by all interaction events in the CdTe that deviate from a full-energy absorption, which greatly simplifies the procedure. For the automated recognition of the peak centroid, a simplified approach relying on automated search of Gaussian peaks is used (Python library module: SciPy) where the user must define some parameters that facilitate the automated recognition of the peaks. This simplification does not take into account incomplete charge collection that is known to affect this type of semiconductor based detectors [5, 8, 9] and that is energy-dependent. However, the effect of this simplification is small and limited to a maximum deviation of 0.4 keV [8] at the largest energies of interest in X-ray dosimetry for radiation protection, and, in line with the scope of this work of providing a practical method to implement the ISO 4037:2019 standard. The energy calibration is provided as output and a linear function was chosen. The same calibration spectra were used to measure the full width at half maximum (FWHM) of the CdTe detector in the energy range from 10 keV to 140 keV. A Gaussian fit was performed on the selected peak and the standard deviation obtained as fit parameter was used to obtain the peak’s FWHM using the relation *FWHM* = 2*.*35 · *σ* [10]. In some cases, multiple peaks are very close (e.g. 79.6142 keV and 80.9979 keV for ^133^Ba [11], for example) and the use of a Gaussian fit function will lead to an incorrect standard deviation; in these cases, the used peak’s fit function was a sum of two Gaussians with the same standard deviation but different centroids. To fit the FWHM in function of the peak’s energy the function used is $HM\ \left[ keV\right]=a+\sqrt{b\bullet E\ \left[ keV\right]}$ . Results are shown in Fig. 1. This analysis was done using SciPy Python’s library module.

### Experimental set-up

**Figure 2:**
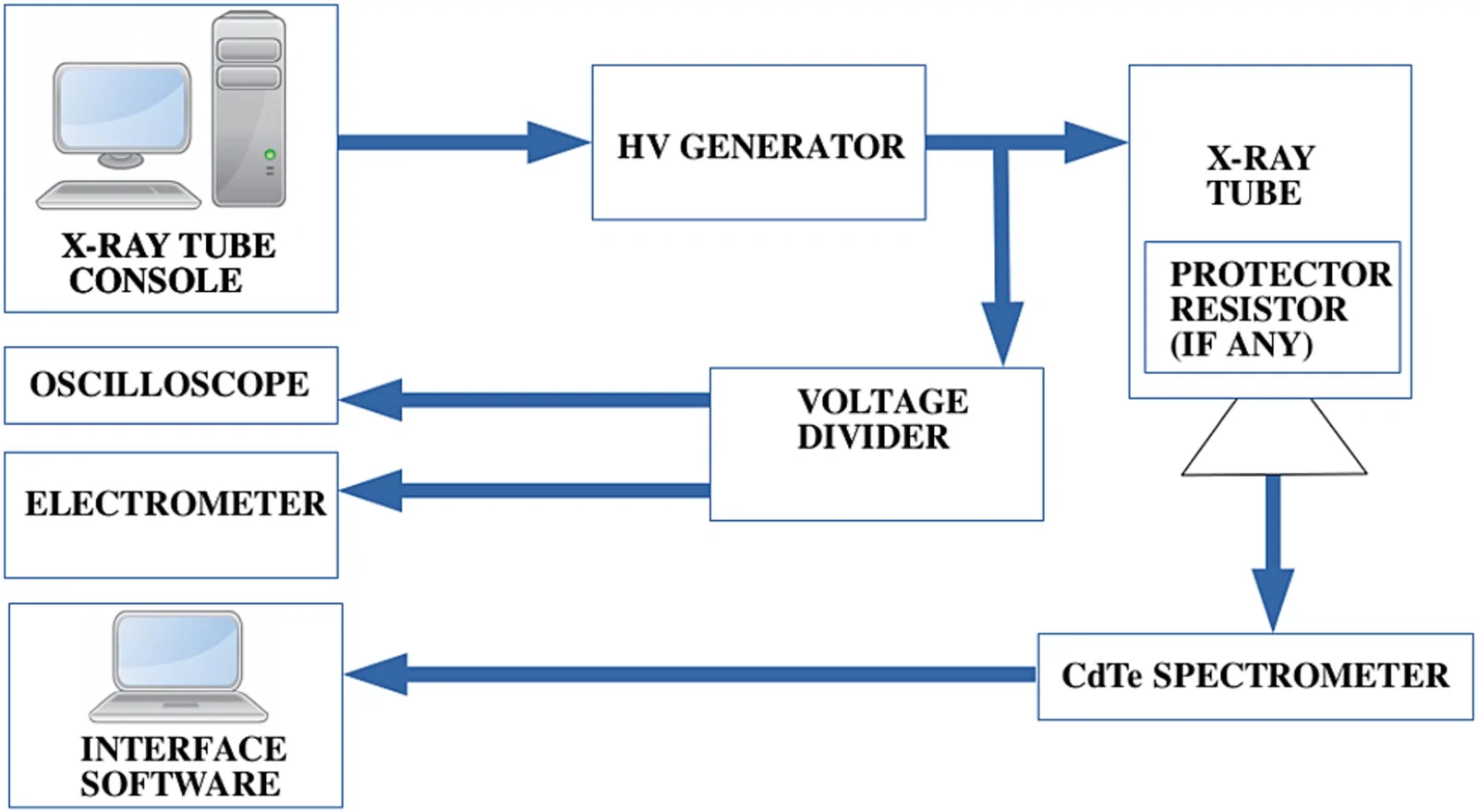
Graphic layout of the experimental set-up.

For each described X-ray tube apparatus, we used the experimental setup as reported in Fig. 2. Our measurements are geared toward characterizing the output of the tubes with validation provided not only by the dividers but especially by the spectrometer. As it is well known, all the constant voltage power supplies, either linear or switching-mode, present a constant voltage mean value with superimposed small oscillations produced in the rectifying process, with a frequency of hundreds of Hz or tens or hundreds of kHz. These variations were experimentally investigated by means of oscilloscope measurements and estimated in order to understand their effects on the position of the spectrum energy endpoint. The distance of the CdTe detector from the focus was increased with higher tube currents in order to reduce the fluence of incident photons, and thus the pile-up. A measurement of the cathode current is inferred from a voltage measurement that is taken from a circuit board inside the power supply of the X-ray tube. Throughout all experimental sessions, the digital console (reporting the actual measured current rather than the nominal set-point) showed no observable fluctuations. In light of these technical characteristics, the uncertainty associated with the tube current was considered negligible. For all qualities studied at ENEA and STUK we added filtration (aluminium, tin, copper, and lead) to standard qualities in order to reduce the fluence and decrease the electronic pile-up. The filters applied modify the shape of the spectrum as they selectively cut the low energy part (beam hardening) but the objective of this study is not to determine the shape of the fluence spectrum, but rather its energy endpoint. Thus, the pulse-height spectra, obtained from the filtration, are relatively narrow and present a steep descent in the terminal part of the spectrum. The beam hardening, achieved with the additional filters, implies a reduction of the photon fluence, which means a lower dead time and a negligible electronic pile-up from the CdTe detector point of view. Finally, using the SpekPy toolkit [12], different fluence spectra were simulated to study the variation of the conversion coefficient ${h}_K^{\ast }$ with respect to the potential between anode and cathode voltage divider reading and corrected value from spectrometry.

#### Endpoint energy estimation

**Figure 3:**
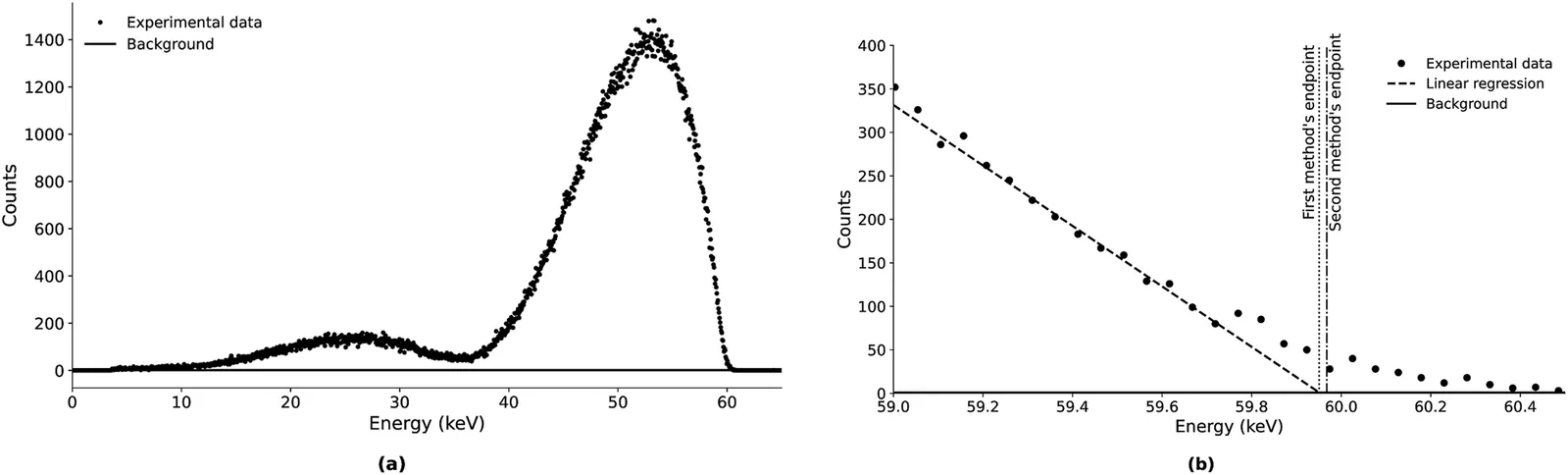
(a) Raw spectrum at 60 kV (N60 X-ray beam quality). (b) Exploded view of the high-energy region of the same raw spectrum (a) showing the endpoints obtained by applying the two methods.

The endpoint energy was determined from the measured pulse-height spectrum using two complementary methods. In the first method, the background radiation level is assumed to be approximately constant. The spectrum endpoint is identified as the intersection between a linear fit of the high-energy tail and the horizontal line representing the mean background. A typical problem with this method is the choice of the ideal energy range in which to perform the linear regression. The linear regression is first performed in the energy range (*A*_min_, *A*_max_) where *A*_max_ is an energy value near the endpoint energy and *A*_min_ *< A*_max_ such that there are at least 10 energy bins between *A_min_* and *A_max_* and the *R*^2^ coefficient of the linear regression is stored. Several iterations are made varying *A*_min_ moving one step toward low energy. The whole procedure is repeated by varying the value *A*_max_. The selected energy range was chosen so to maximize the *R*^2^ coefficient, spectrum tail and linear fit in the selected energy range are plotted so that the operator can check the results. The second method, instead, is based on the estimation of the measured background and the energy resolution of the detector. An estimation of the mean background counts (*C_b_*) is obtained from the measured background spectrum; then an algorithm starting from the spectrum high energy region searches the first channel with a number of counts (*C*) such that *C >* 3*C_b_* + 3. We have chosen the value 3*C_b_* because we assume the background counts have a Gaussian distribution with *C_b_* as mean value. Knowing the standard deviation, we find that the value 3*C_b_* has a probability to occur *<*0*.*01% to lie within the background. In this way we are confident that the number of counts *C >* 3*C_b_* are generated by photons from the X-ray tube. At this point, the peak broadening caused by spectrometer energy resolution must be considered. Applying the second method to a mono-energetic peak, assuming the peak has a Gaussian shape, it leads to a value on the high energy tail instead of its centroid. To bring the estimated endpoint to the centroid, knowing the FWHM(E) as function of the energy, a value equal to FWHM(E)/2 is subtracted from the obtained high energy tail value. In this way, we correct the energy endpoint estimated with the second method. In the present work, the endpoint is evaluated using both methods, yielding two distinct values, as shown in Fig. 3 for the case of 60 kV (N60 as in ISO 4037 [1]). Since there is no pre-knowledge regarding the probability distribution associated with the endpoint energy, a conservative approach was chosen based on a uniform-type distribution. Since the two methods are consistently defining an interval, with one method always over-estimating the other, the values obtained from the two methods are used as extremes of the uniform distribution. We assume that the real value of the endpoint energy is uniformly distributed within the range of these estimated values, and we obtain the final endpoint energy as the mean value of this distribution with its standard deviation.

## Results

### HV generators ripple estimation

As already mentioned in the previous section, voltage power supplies present a constant voltage mean value with superimposed small oscillations from rectifying processes, with a frequency of hundreds of Hz or tens or hundreds of kHz. These voltage oscillations are in the range of kV, varying the voltage difference between anode and cathode. These variations were experimentally investigated and estimated in order to understand their effects on the position of the energy spectrum endpoint. It has been found out, by an oscilloscope, that the ripple oscillations of the positive voltage supply connected to the anode are equal to and in phase with the ripple oscillations of the negative voltage supply connected to the cathode. This is possible because the two voltage power supplies are switching-mode with synchronized inverter oscillators at 17 kHz. Therefore, the two ripples cancel each other out. However, the two ripple signals possessed also several overshoots at several hundreds of kHz which are conversely in phase opposition and hence they do not cancel out but they add up instead, and, together with the HV mean values, can produce a slight increase of the HV difference and hence shift the spectrum endpoint to slightly higher energies. Whilst the ripple period is ~60 μs, the duration of the overshoots is ~2 μs. This means that the upward parts of the overshoot ringing last ~1 μs or a bit less. Because of this short time, the question was raised as to whether the electrons that fly from the cathode to the anode have the time be affected by these voltage overshoots. An easy estimation of the time of flight of the electrons which are accelerated by the voltage difference between the anode and the cathode showed that this time of flight is in the order of few nano seconds. Therefore, the electrons are indeed affected by these voltage overshoots which make their kinetic energy increase and thus can cause a shift of the spectrum endpoint. The height of these overshoots was measured by an oscilloscope and turned out to vary between 300 and 500 V. Besides, the temporal weight of the overshoots was also estimated by comparing their duration with the ripple period in order to have an idea of how much these “over accelerated” electrons may contribute to shift the energy endpoint of the X-ray spectrum. In the end it appears that the possible shift of the endpoint toward higher energies lies within the uncertainty in determining the endpoint position.

### Voltage calibration and protective resistor

The results, obtained with the methods described in the previous section, provide two strategies for the HV calibration of a X-ray tube both with and without the protective resistor.

**Figure 4:**
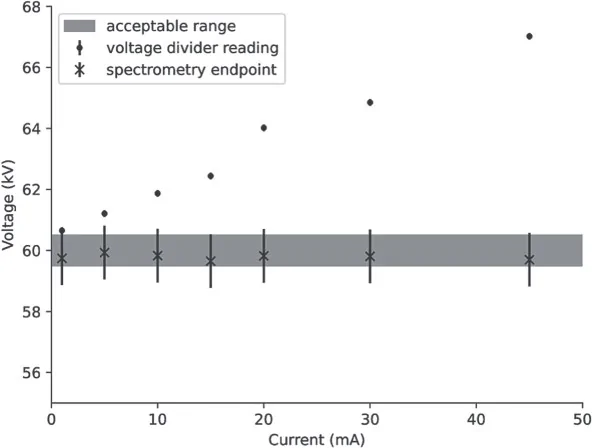
Data obtained from ENEA-INMRI voltage dividers and CdTe spectrometer as function of current for 60 kV nominal voltage case. The voltage values measured with voltage dividers are indicated as points with their uncertainties of 0.25% (*k =* 1). The endpoint energy values calculated from spectrometry measurements are shown as crosses with their uncertainties of 1.5% (*k =* 1), see *Uncertainty budget* Section. The grey band represents the acceptable range criteria.

The first scenario refers to the X-ray tube equipped with the protective resistor (ENEA-INMRI X-ray tube). In this case, the endpoint energy evaluated from the measured X-ray spectrum is the proposed method to determine the high voltage difference applied between anode and cathode. As an example, the results for the nominal voltage of 60 kV are plotted in Fig. 4 and reported in Table 1. Figure 4 shows the voltage values measured with voltage dividers and the endpoint energy values as function of current. It can be observed that to achieve a certain tube voltage value of 60 kV, we compensate the tube voltage setting on the console so that the measured endpoint falls within the acceptable range around the desired potential.

**Table 1:** Values for 60 kV nominal voltage with the ENEA-INMRI X-ray tube equipped with the protective resistor.

Anode current (mA)	Voltage divider		Endpoint	
	Reading (kV)	Uncertainty k = 1 (kV)	Reading (kV)	Uncertainty k = 1 (kV)
1	60.7	0.1	59.7	0.9
5	61.2	0.1	59.9	0.9
10	61.9	0.1	59.8	0.9
15	62.4	0.1	59.6	0.9
20	64.0	0.1	59.8	0.9
30	64.9	0.1	59.8	0.9
45	67.0	0.1	59.7	0.9

The data are shown in Fig. 4.

The acceptable range was chosen to be ±0.5 kV.

**Figure 5:**
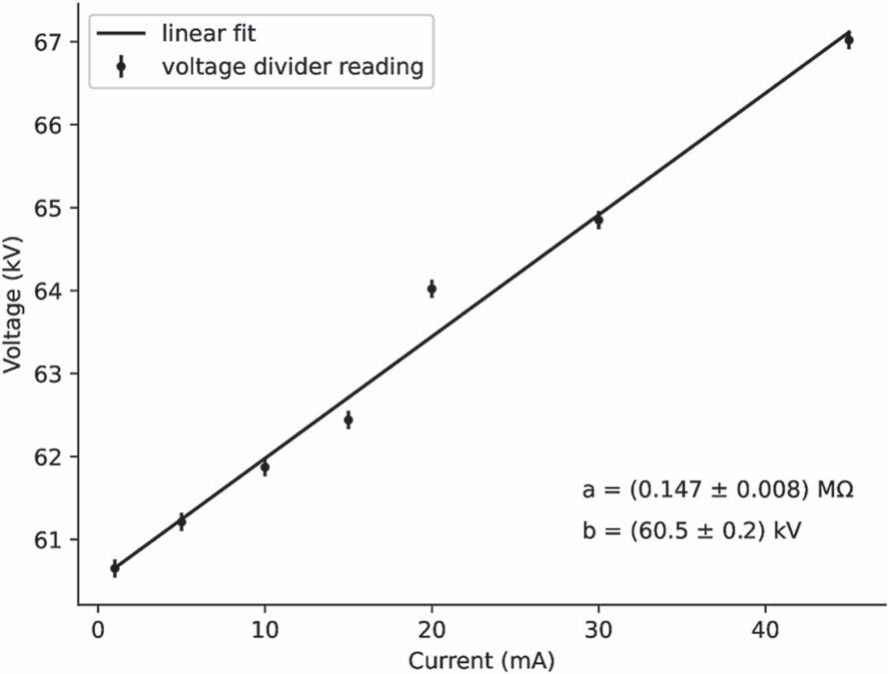
Linear fit of ENEA-INMRI voltage divider reading compensated by the potential drop due to the protective resistor for 60 kV desired voltage. Experimental data and their uncertainties are presented in Table 1.

From spectrum endpoint estimations, the tube voltage was stable within 0.3 kV whilst the voltage divider result varied from 60.7 kV to 67.0 kV. This means that if one is not aware of the presence of the tube protective resistor, a significant voltage difference may be applied between cathode and anode as the current increases (e.g. 10% at 45 mA for 60 kV). Spectrometry provides a downstream measurement of the X-ray output which is not accessible to a measurement using a voltage divider. Applying a linear fit *a* · *x* + *b* to the voltage divider data as a function of the current, the slope of the best-fit line estimates the protective resistance value and this parameter is provided with an uncertainty due to the linear fit, Fig. 5. The protective resistor value for this tube is estimated to be ~150 *k*Ω, in reasonable agreement with the approximate value of 100 *k*Ω indicated in the ISO 4037 [1]. Knowing the value of the protective resistance *R*, we are able to quantify the potential drop and compensate the console settings for it in order to obtain the desired voltage *V*_nominal_ expressed in kV between anode and cathode, using this simple relation


**Figure 6:**
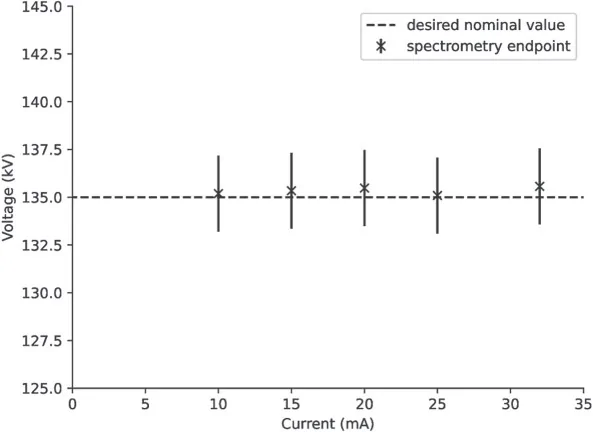
Extrapolated data analysis from ENEA-INMRI X-ray tube, thanks to spectrometer method, as a function of current for 135 kV desired voltage. The uncertainties reported in the plot are the spectrum endpoint total uncertainties (*k =* 1), see *Uncertainty budget* Section. Dotted line represents the desired voltage value.

(1) \begin{eqnarray*} {V}_{divider}={V}_{nominal}+R\bullet c \end{eqnarray*}

where *V*_divider_ refers to the voltage dividers measurement, *R* is the resistance, and *c* is the current. To verify the estimated resistance value, we repeated the measurements at a different nominal voltage. The voltage selected is 135 kV because, due to the power limits of ENEA-INMRI generators, moving to larger voltage values could limit the current range which, in fact, we wish to keep as large as possible to demonstrate the effectiveness of the approach. We use equation 1 to calculate the value *R* · *c* in order to obtain *V*_divider_ for a fixed desired voltage value between anode and cathode. The results are shown in Table 2 and plotted in Fig. 6. The obtained voltage values, reported in Table 2, were stable within 0.5 kV whilst the voltage divider result varied from 135.1 kV to 138.3 kV. This demonstrates that the tube potential is stable with a very low deviation.

**Table 2:** Values for 135 kV nominal voltage with a X-ray tube equipped with the protective resistor.

Anode current (mA)	Voltage drop R*∙*c (kV)	Endpoint (kV)	Endpoint uncertainty k = 1 (kV)
10	1.5	135.2	2.0
15	2.2	135.3	2.0
20	2.9	135.5	2.0
25	3.7	135.1	2.0
32	4.7	135.6	2.0

The data are shown in Fig. 6.

**Figure 7:**
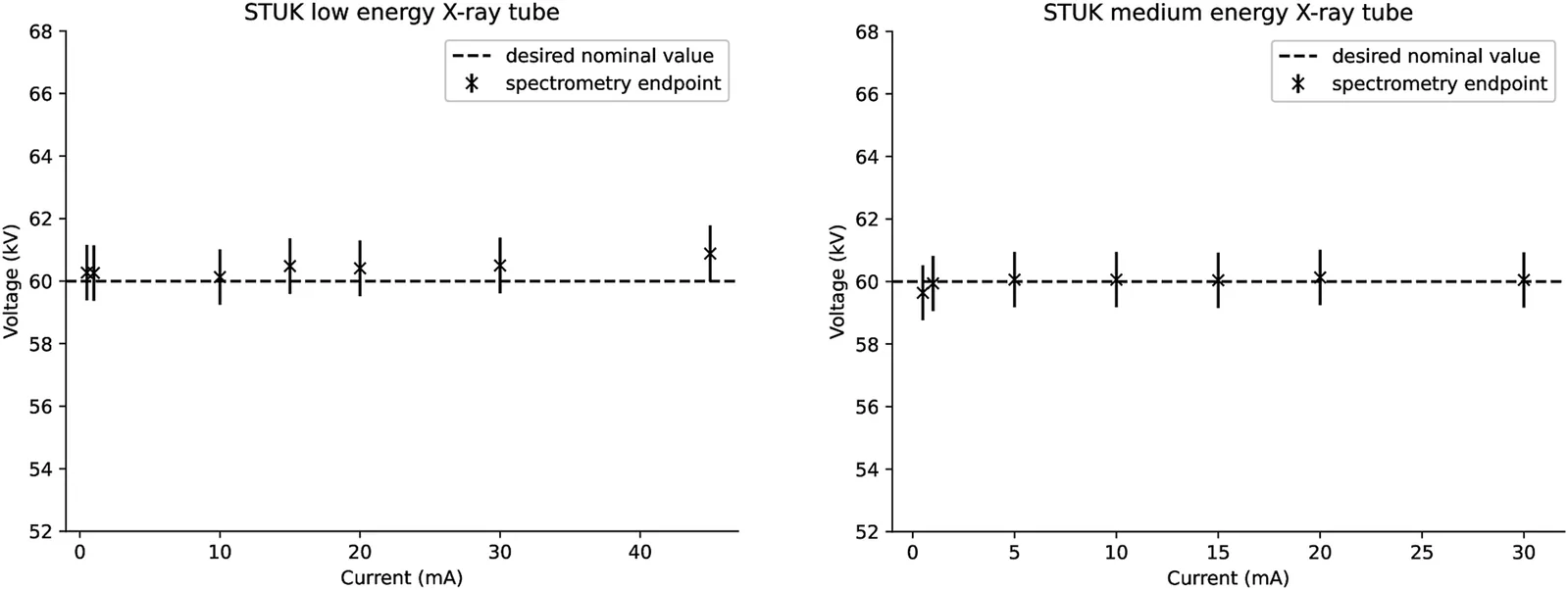
Top, results from STUK low energy X-ray tube. Bottom, results from STUK medium energy X-ray tube. The uncertainties reported in the plots are the spectrum endpoint total uncertainties (k = 1), see *Uncertainty budget* Section.

These results show that the estimated resistance value is consistent and, using equation 1, we can predict the voltage divider reading to be set to obtain the desired voltage value that is effectively applied between tube anode and cathode. Similar measurements were performed in July 2023 at STUK. As described in the previous sections, both X-ray systems have no protective resistor and no voltage dividers are permanently installed in the facility. The voltages for both X-ray tubes were set close to 60 kV. Voltage values were fixed, and for the ENEA-INMRI X-ray tube the anode current varied in the range 0.5–30 mA, and for the low-energy tube from 0.5 mA to 45 mA. In Fig. 7, it can be seen that the generating potential, obtained with the spectrometry method, does not depend on the current for both tubes. Considering the ENEA-INMRI X-ray tube, the obtained voltage values were stable within 0.5 kV, whilst for low energy tube, the obtained voltage values were stable within 0.7 kV. The results confirm that no protective resistor is installed in either STUK X-ray tubes, and the generating potential and tube potential are consistent and independent of tube current. The endpoint values obtained by the presented method are in agreement, within the uncertainty, with those obtained by STUK.

### Uncertainty budget

An analysis of uncertainty components was performed, following the international guidance [13]. Here, all uncertainties presented are relative standard uncertainties, at coverage factor k = 1. Each ENEA-INMRI calibrated voltage divider system has an estimated uncertainty of 0.25% (k = 1). Since the ENEA-INMRI X-ray tube has two generators we need two voltage dividers, each one with its calibration coefficient and its electrometer, then the total uncertainty is given by using the general formula for uncertainty propagation. Analysis of the influence on uncertainty of the spectrometer energy calibration curve and energy resolution was performed by varying the number of the X and *γ* lines considered in the calibration and energy resolution estimation. Spectra from the radioactive sources were combined in a set of 2, 3, or 4 sources, in order to obtain between 4 and 7 lines for the calibration and energy resolution estimation.

Using the energy calibrations performed over 2 y, it was possible to assess the uncertainty due to the temporal stability of the energy calibration procedure. Several background spectra were measured in order to use them to estimate the uncertainty due to the background on the estimated endpoint energy. The proposed method for estimating the spectrum endpoint energy was applied using these background spectra, and this component of the uncertainty was thus estimated. To estimate the uncertainty related to Method 2, endpoints are obtained by first choosing a smaller and then a larger channel than the one selected by the algorithm and the semi-range between these two values provides an estimate of the uncertainty. The Monte Carlo method was used to estimate the uncertainty associated to Method 1 and counting statistics according to the Guide for expression of Uncertainty in Measurements [14]. It consists of varying each quantity within its uncertainty 10 000 times and estimating the endpoint energy each time to obtain the effect of variation of the endpoint energy value. The distribution of endpoint energy values gives a Gaussian shape and its width obtained by a fit procedure is taken as uncertainty for the component under consideration. Table 3 summarizes the values obtained for each uncertainty component. All uncertainty components are assumed to be uncorrelated and are summed in quadrature to obtain an estimate of the combined standard uncertainty. The resistor value was obtained using a linear least-square fitting procedure with a linear function on voltage divider readings with their uncertainties as function of current. The fit parameters were estimated with their uncertainties [15] and the resistor uncertainty ${u}_R$ is 2% (k = 1). From equation 1, using the general formula for relative uncertainty propagation, we can obtain the desired voltage ${V}_{nominal}$ uncertainty in presence of protective resistor.

**Table 3:** Uncertainty budget related to the endpoint energy determination at k = 1.

Source of uncertainty	Relative uncertainty (%)
Energy calibration stability in time	0.18
Energy calibration curve	0.12
Energy resolution (FWHM)	0.44
Background	0.08
Counting statistic	0.07
Method 1	1.33
Method 2	0.17
Rectangular distribution	0.33
**Combined standard uncertainty**	**1.5**

### Implications on conversion coefficients $\boldsymbol{{h}_K^{\ast }(10)}$

**Figure 8:**
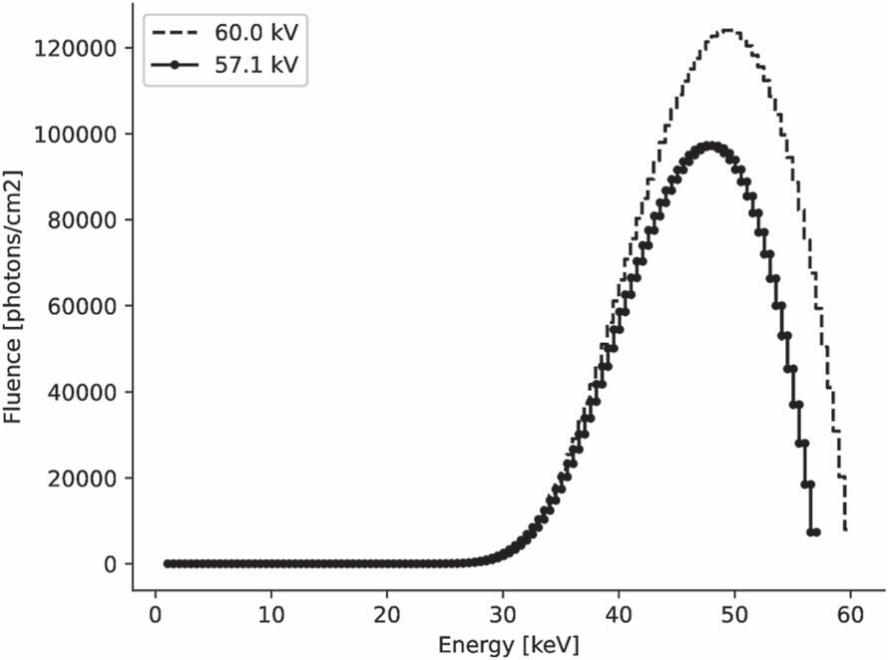
SpekPy N-60 simulated spectra for different tube potentials.

The voltage characterization of an X-ray system has an influence on the determination of radiation qualities and conversion factors for calibration services. A sensitivity analysis of the conversion coefficients from air kerma to the quantity ambient dose equivalent H^*^(10) is presented below to understand whether the accuracy of spectrometry-based measurements of spectral endpoint energy is sufficient for radiation protection dosimetry applications. Starting from a ionization chamber calibrated in terms of air kerma *K*_a_, one can obtain a measurement of H^*^(10) using the conversion coefficients. The simulated X-ray tube used in SpekPy has a tungsten target with a 20° anode angle and a 3 mm thick beryllium window. N-60 filter was composed by 4.02 mm of aluminium and 0.61 mm of copper. The spectrometer was positioned 100 cm from the focus along the beam axis. To allow for air attenuation, an additional filter made of air with thickness of 100 cm was added. Two fluence spectra were simulated: in the first simulation we assumed no protective resistor in the tube and we set the tube potential equal to the voltage divider readout of 60 kV; in the second case we took into account a lower high voltage value, consequence of the voltage drop explained and demonstrated earlier, occurring in an X-ray tube with a protective resistor. We experimentally found the value of the protective resistor and the potential between anode and cathode was calculated using equation 1. As we discussed in the previous paragraph, when the divider read-out is 60.0 kV, the effective potential measured is 57.1 kV for a tube current of 20 mA. This two voltage values are the tube potentials chosen for the comparison. Simulated spectra are shown in Fig. 8. Spectrum averaged values of the conversion coefficients were calculated by simulated fluence spectra with the corresponding conversion coefficient from *K*_a_ to H^*^(10) for mono-energetic photons h^*^_K_(10;*E*), as tabulated in ISO 4037:2019–3 chapter 6.3 [3]. The conversion coefficient ${h}_K^{\ast}\left(10;N\right)$ for the narrow series N-60 is obtained using the following equation (2):


(2) \begin{eqnarray*} {h}_K^{\ast}\left(10;N\right)=\frac{\int_{E_{min}}^{E_{max}}{\Phi}_E(E)\bullet{k}_{\Phi}(E)\bullet{h}_K^{\ast}\left(10;E\right) dE}{\int_{E_{min}}^{E_{max}}{\Phi}_E(E)\bullet{k}_{\Phi}(E) dE} \end{eqnarray*}

where ${k}_{\Phi}(E)=E\bullet \frac{\mu_{en}(E)}{\rho }$represents the energy dependent kerma factors, with mass energy-absorption coefficient $\frac{\mu_{en}(E)}{\rho }$ values for mono-energetic photons in dry air taken from Hubbell *et al.* [16] and interpolated between two points, Φ*_E_*(*E*) is the fluence, whilst *E*_min_ and *E*_max_ are the energy limits of the adopted fluence spectra. The calculated values of ambient dose equivalent for each of the two spectra are reported in Table 4.

**Table 4:** Conversion coefficient, ${h}_k^{\ast }(10)$, in case of nomina potential and a lowered potential by 2.94 kV and the percentage difference between the them.

ISO quality	${h}_k^{\ast }(10)$ from voltage divider (Sv/Gy)	${h}_k^{\ast }(10)$ corrected (Sv/Gy)	Percentage difference (%)
N-40	1.18	1.12	5.64
N-60	1.58	1.56	1.54
N-80	1.73	1.72	0.04
N-100	1.72	1.72	0.02
N-120	1.72	1.72	0.00
W-60	1.49	1.47	1.70
W-80	1.64	1.64	0.52

The values were calculated for several X-ray qualities using simulated spectra by means of SpekPy.

The conversion coefficients differ by 1.54% for the two simulated spectra. Simulations for several X-ray ISO 4037:2019 qualities implemented in our laboratories were performed for a sensitivity analysis of the conversion coefficients. A current of 20 mA was assumed for all qualities, leading to a fixed compensation of 2.94 kV as in equation 1, to compensate for the presence of the protective resistor. Conversion coefficients were calculated for each quality from the simulated spectra obtained by first using the potential difference read by the voltage divider and then using that obtained from spectrometry; results are summarized in Table 4. The corrections are relevant mostly for low potential values, indeed a significant difference higher than 4% is observed for the ISO quality N-40. The effect is larger with lower voltage qualities. The reason for this is the sensitivity of the spectrum shape to small changes in the voltage and the large gradient of monoenergetic conversion coefficients at low energies, as provided in [3]. The data we presented here are an example and we did not check the effect of voltage drop below 40 kV generating potential since this is the lowest high voltage value in use at ENEA-INMRI with the 450 kV.

X-ray tube under investigation. In addition, we chose a unique 20 mA tube current value because this value allows calibrations to be performed in a reasonably short time and it’s a typical current value used in measurements with several instruments. In light of these results, the majority of the estimated values of the conversion coefficients h^*^_K_ are in agreement within an uncertainty of 4% for k = 2 with the values reported in the ISO 4037:2019 standard. The presence of the protective resistor does not significantly modify the values of the coefficients for the N-series qualities with tube voltage higher than 60 kV. For the N-40 quality, the presence of the protective resistor introduces an uncertainty ˃4%, so that, in this case, it is essential to know of the presence of a protective resistor to obtain the effective potential difference between anode and cathode.

## Conclusion

One of the questions facing the community of ionizing radiation metrology institutes at this time, be these Primary or Secondary Standards Laboratories, is if spectrometry could be accepted in the future in place of calibrated voltage dividers as the recommended method to determine the X-ray tube potential according to the ISO 4037:2019 standard. The determination of the spectrum endpoint energy has been investigated by Šolc *et al.* [4] and many studies are underway on the topic [6, 17] with measurement campaigns set up with different type of spectrometers, voltage dividers, and multimeter dosimetry systems for reference dosimetry in diagnostic radiology (X-ray MultiMeters – XMMs [18]). We have shown here how to determine the tube voltage with a CdTe spectrometer and estimate the value of the resistance of the protective resistor, then using that value to make appropriate corrections to high voltage readings based on voltage dividers. The spectrometry method is surely less expensive and accessible to a larger group of laboratories that cannot afford the purchase and periodic calibration of high voltage dividers. Importantly, in this procedure, the spectrometer is used only for endpoint evaluation and so no unfolding data processing is needed [7, 8]. This makes the spectrometry-based approach described here significantly more accessible even to smaller laboratories with a limited experience in spectrometry. We investigated the effect of using an X-ray tube with protective resistor and high voltage dividers on the determination of conversion coefficients from air kerma to H^*^(10) using simulated spectra. Differences ˂4% for most of the considered radiation qualities were observed. Importantly, under the setup described here, the anode current measurement is not traceable, a limit in the approach described. However, given the effectiveness of the corrections to the voltage value that we made based on the determination of the protective resistance, the lack of traceabilty in the anode current measurement did not appear to be critical. Future developments will aim at reducing the uncertainty in the determination of the spectrum endpoint 411 energy and in the application of this method to perform calibration in terms of voltage for XMMs which are largely used for quality control tests in diagnostic radiology [18].
